# PSM-Mec—A Virulence Determinant that Connects Transcriptional Regulation, Virulence, and Antibiotic Resistance in Staphylococci

**DOI:** 10.3389/fmicb.2016.01293

**Published:** 2016-08-22

**Authors:** Li Qin, Joshua W. McCausland, Gordon Y. C. Cheung, Michael Otto

**Affiliations:** ^1^Pathogen Molecular Genetics Section, Laboratory of Bacteriology, National Institute of Allergy and Infectious Diseases, National Institutes of HealthBethesda, MD, USA; ^2^Department of Dermatology, Wuhan No. 1 Hospital, Tongji Medical College, Huazhong University of Science and TechnologyWuhan, China

**Keywords:** *Staphylococcus aureus*, *Staphylococcus epidermidis*, phenol-soluble modulin, PSM-mec, regulatory RNA, SCC*mec*, virulence

## Abstract

PSM-mec is a secreted virulence factor that belongs to the phenol-soluble modulin (PSM) family of amphipathic, alpha-helical peptide toxins produced by *Staphylococcus* species. All known PSMs are core genome-encoded with the exception of PSM-mec, whose gene is found in specific sub-types of SCC*mec* methicillin resistance mobile genetic elements present in methicillin-resistant *Staphylococcus aureus* and coagulase-negative staphylococci. In addition to the cytolytic translational product, PSM-mec, the *psm-mec* locus encodes a regulatory RNA. In *S. aureus*, the *psm-mec* locus influences cytolytic capacity, methicillin resistance, biofilm formation, cell spreading, and the expression of other virulence factors, such as other PSMs, which results in a significant impact on immune evasion and disease. However, these effects are highly strain-dependent, which is possibly due to differences in PSM-mec peptide vs. *psm-mec* RNA-controlled effects. Here, we summarize the functional properties of PSM-mec and the *psm-mec* RNA molecule and their roles in staphylococcal pathogenesis and physiology.

## Introduction

*Staphylococcus aureus* is a serious human pathogen responsible for a multitude of human diseases, which range from acute skin and soft tissue infections to more severe illnesses such as catheter-associated bacteremia, necrotizing pneumonia, and osteomyelitis. Since the emergence of methicillin-resistant *S. aureus* (MRSA) in the early 1960s, *S. aureus* has continued to cause significant morbidity, mortality, and a considerable financial burden for public health systems. For *S. aureus* and its closely related cousin, *Staphylococcus epidermidis*, the acquisition of antibiotic resistance, coupled with the ability to attach to surfaces and form sticky, multicellular agglomerations (biofilms; Costerton, 1999) on indwelling catheters, are two defining hallmarks that account for the ongoing difficulty in treating immuno-compromised individuals (Otto, 2008) with infections caused by Hospital-Associated MRSA [HA-MRSA] (Otto, 2012) and *S. epidermidis* (Otto, 2009).

Genetic mutations and/or the acquisition of mobile genetic elements (MGEs) that harbor drug resistance genes are two common mechanisms that are responsible for antibiotic resistance in microorganisms. Arguably the most important staphylococcal MGE is *Staphylococcus* cassette chromosome *mec* (SCC*mec*), which codes for methicillin resistance. Recently, a unique locus was discovered in specific types of SSC*mec* elements in *S. aureus* and *S. epidermidis*, which comprises a small regulatory (sr)RNA and an embedded gene encoding a cytolytic peptide called PSM-mec (Queck et al., 2009). PSM-mec belongs to the phenol-soluble modulin (PSM) family of amphipathic, alpha-helical peptide toxins produced by *Staphylococcus* species (Wang et al., 2007, 2011; Diep and Otto, 2008; Otto, 2014; Cheung et al., 2014a), which collectively play an important role as virulence determinants in many facets of *S. aureus* and *S. epidermidis* pathogenesis (Wang et al., 2011; Periasamy et al., 2012; Cassat et al., 2013; Cheung et al., 2014a). Here, we will discuss the current knowledge on the PSM-mec and the *psm-mec* srRNA molecule, with a focus on their roles in *S. aureus* pathogenesis.

## The distribution of the *psm-mec* locus in SCC*mec*

**Figure 1:**
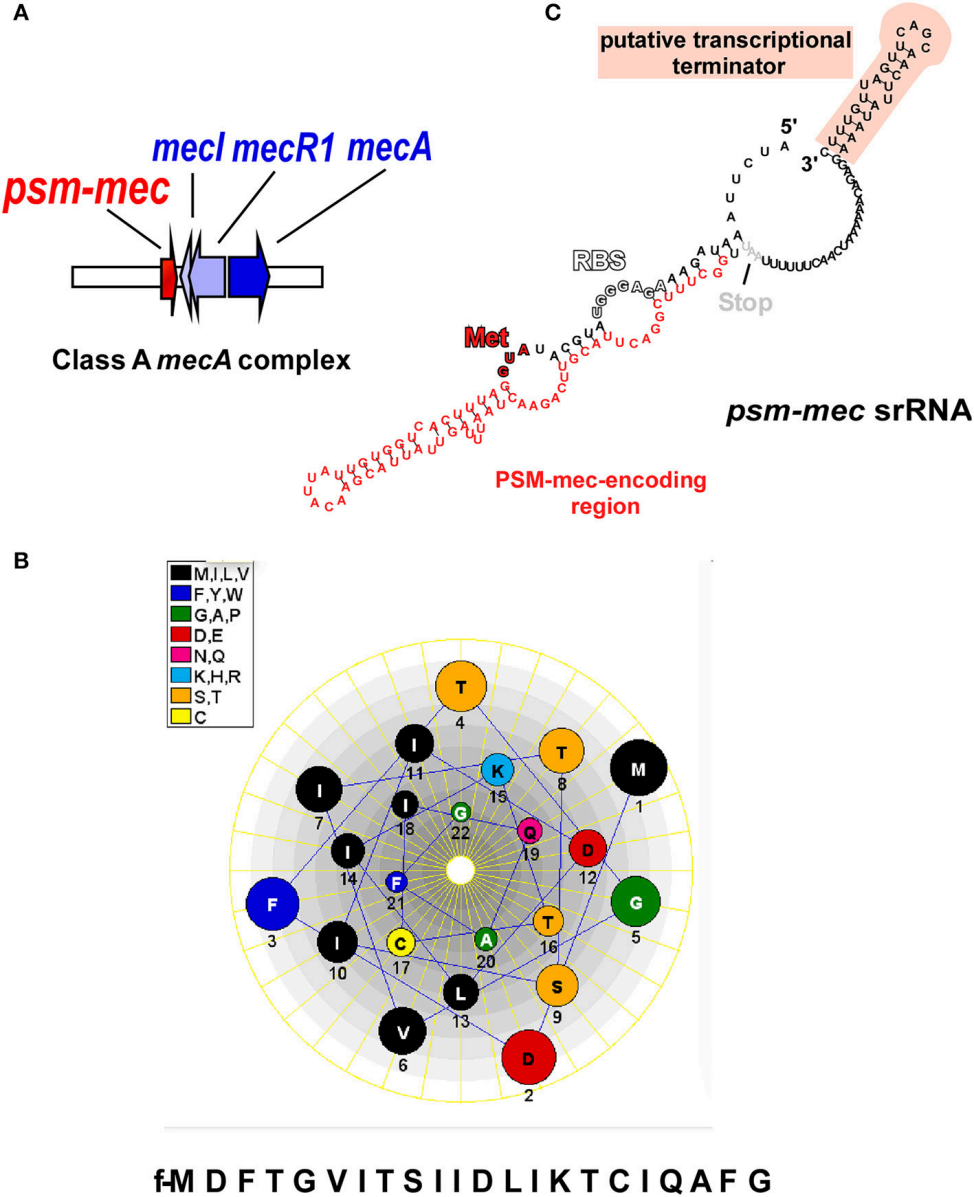
**The ***psm-mec*** locus and its products. (A)** The *psm-mec* gene is located on the class A *mecA* complex in SCC*mec* elements of types II, III, and VIII, next to the *mecI*/*mecR1*/*mecA* genes that confer methicillin resistance and its regulation. **(B)** The PSM-mec peptide product of the *psm-mec* locus forms an α-helix with pronounced amphiphathy; helical wheel presentation. **(C)** The *psm-mec* gene is part of a regulatory RNA with pronounced predicted secondary structure. The *psm-mec* gene is entirely embedded in a highly folded, large stem loop. The *psm-mec* srRNA only slightly extends toward the 3′ end and ends with a characteristic predicted terminator stem loop structure (Note, however, that Kaito et al. reported the srRNA to be slightly longer at the 5′ end). RBS, ribosomal binding site; Start codon coding for (N-formyl) methionine.

SCC*mec* elements, which range in size from 21 to 67 kb, comprise as characteristic components, the *mecA* gene that codes for a penicillin-binding protein, a *ccr* gene complex for site-specific recombination, and flanking repeat sequences for integration into the genome (International Working Group on the Classification of Staphylococcal Cassette Chromosome Elements, 2009). SCC*mec* elements are classified into 11 different “types,” as defined by the presence of different *ccr* and *mec* resistance genes (International Working Group on the Classification of Staphylococcal Cassette Chromosome Elements, 2009; Li et al., 2011; Shore et al., 2011) and further divided into “subtypes” based on the presence of other resistance genes and transposons in the non-essential J regions (Ito et al., 2003). The *mec* gene complex has four core elements; IS*431, mecA, mecR1*, and *mecI*, which represent the genes coding for insertion sequence IS*431*, a modified penicillin binding protein 2a (PBP2a), the signal transducer protein, and the methicillin resistance regulatory protein (International Working Group on the Classification of Staphylococcal Cassette Chromosome Elements, 2009; Shore and Coleman, 2013; Figure 1A).

The *psm-mec* locus is found in the J2 region adjacent to *mecI* of the class A *mec* gene complex in several widespread HA-MRSA lineages, but most prominently in Sequence Type (ST) 5, ST36, ST45, ST225, and ST239 strains (Deurenberg and Stobberingh, 2009; Nübel et al., 2010; Castillo-Ramirez et al., 2012; Schulte et al., 2013), which all have either SCC*mec* types II or III (Chatterjee et al., 2011; Monecke et al., 2012; Josten et al., 2014). However, more detailed analysis revealed that the *psm-mec* locus is found in SCC*mec* types II, IIA, IIB, IID, III, and VIII of many *Staphylococcus* species (Monecke et al., 2012; Shore and Coleman, 2013).

Antibiotic resistance in bacteria is often associated with a reduced fitness cost to the bacterial host (Otto, 2013a). We recently reported that the expression of the PSM-mec peptide impacted oxacillin resistance in *S. aureus* (Cheung et al., 2014b), suggesting that a delicate balance may exist between virulence gene expression and methicillin resistance in MRSA strains carrying the *psm-mec* locus.

## Expression and regulation of the PSM-mec peptide

The production of the PSM-mec peptide, which can be identified in bacterial culture filtrates by high-pressure liquid chromatography/mass spectrometry (HPLC/MS) (Queck et al., 2009) or Matrix-assisted laser desorption/ionization Time of Flight (MALDI-TOF) analysis (Josten et al., 2014), is a reliable indicator of methicillin resistance. However, the restricted distribution of the *psm-mec* locus severely limits the potential use of HPLC-based detection of PSM-mec for routine clinical screening of methicillin resistance.

The expression of all PSMs including the MGE-encoded PSM-mec is positively regulated by the accessory gene regulatory (*agr*) quorum sensing system (Novick, 2003; Queck et al., 2008; Chatterjee et al., 2011). This up-regulation is dependent on the binding of activated AgrA, the response regulator of *agr*, to promoter sequences upstream of the *psm* genes (Queck et al., 2008), but this has not directly been demonstrated for the *psm-mec* promoter. Interestingly, PSM expression is also enhanced in the presence of calf serum (Oogai et al., 2011) and up-regulated within intracellular environments after the activation of the stringent response (Geiger et al., 2012). Whether this is also the case for PSM-mec is not known.

PSM-mec production was shown to be especially frequent among clinical isolates of *S. epidermidis* compared to isolates from the skin of healthy individuals (Queck et al., 2009), which is a direct consequence of the genetic connection of *psm-mec* with methicillin resistance, which occurs much more frequently in clinical isolates. However, expression levels of PSM-mec can vary strongly among isolates (Queck et al., 2009). The mechanistic reasons for varying levels of PSM-mec expression between isolates cannot be fully explained but a documented -7T > C mutation in the *psm-mec* promoter is known to diminish PSM-mec expression in a subset of HA-MRSA strains (Kaito et al., 2011, 2013; Aoyagi et al., 2014).

## Characterization of PSM-mec peptide

Like all members of the PSM family, PSM-mec is expressed with an N-terminal formyl-methionine and secreted without a signal peptide (Wang et al., 2007; Queck et al., 2009). Although this has not yet been shown directly for PSM-mec, it is likely that it is also exported by one or both of the two dedicated ATP-Binding Cassette (ABC) transporters that secrete PSMs (Chatterjee et al., 2013; Yoshikai et al., 2016). The 22 amino acid PSM-mec fits into the class of shorter α-type PSMs, which are anywhere between~ 20–25 amino acids in length, as opposed to the β-type, which are approximately twice as long (43–45 amino acids) (Cheung et al., 2014a).

PSM-mec has a unique cysteine residue at position 17, while cysteine residues are not present in the sequences of any other known PSM (Figure 1B). The notion of a hypothetical dimeric form of PSM-mec, due to the formation of disufide bridges created from oxidized cysteine residues, was formally discounted after size exclusion chromatography experiments showed no difference in extracted ion chromatograms of PSM-mec mutants compared to that of the unaltered PSM-mec peptide (Queck et al., 2009). However, the cysteine residue was crucial for pro-inflammatory and cytolytic activity, probably because it is essential for the PSM-mec secondary structure (Queck et al., 2009).

## The pro-inflammatory and cytolytic properties of PSM-mec

At nanomolar concentrations, the PSMs are powerful pro-inflammatory agents and have an extraordinary capacity for activating and stimulating neutrophils (Wang et al., 2007), which is exerted through their interactions with human formyl peptide receptor 2 (FPR2; Kretschmer et al., 2010), a G protein-coupled receptor expressed on multiple immune cell types (Migeotte et al., 2006). Although differences in activity are observed between PSM members, PSM-mec readily up-regulates both CD11b and gp91phox expression, induces calcium flux, chemotaxis, and IL-8 release in neutrophils (Queck et al., 2009).

On the other hand, several PSM members of the α-type possess potent cytolytic activities, and an ever-increasing number of immune and non-immune cells are being discovered that are subject to PSM-mediated cytolysis (Cheung et al., 2014a). The lysis of eukaryotic cells by PSMs, which occurs at micromolar concentrations, is likely mediated in a receptor-independent manner (Kretschmer et al., 2010), While the involvement of receptors cannot be formally excluded, the facts that PSMs are surfactant molecules, lyse artificial phospholipid vesicles (Duong et al., 2012; Laabei et al., 2014), and FPR2 is not involved in lysis (Kretschmer et al., 2010), speak for such a receptor-independent mechanism. Lytic activity is, however, heavily influenced by membrane composition (Laabei et al., 2014). It is believed to be crucial for PSM-mediated immune evasion and progression of staphylococcal pathogenesis. PSM-mec follows the characteristic cytolytic profile of some α-type PSMs and can lyse human erythrocytes and neutrophils at a comparatively moderate level that is similar to that exerted by δ-toxin (Queck et al., 2009).

## The *psm-mec* srRNA

The first clue for the presence of an srRNA within the *psm-mec* locus stemmed from experiments with MRSA strains transformed with a plasmid carrying 575 nucleotides amplified from SCC*mec* type II, termed the “F region” (Kaito et al., 2008). The authors reported that an open reading frame (ORF) transcribed in the opposite direction of the *psm-mec* gene, coined “*fudoh*,” encoded a protein that controlled colony spreading (Kaito et al., 2008). It was later discovered from experiments using *S. aureus* isogenic *fudoh* point-deletion mutants that the phenotypic differences were in fact influenced by an srRNA (Kaito et al., 2011), which contains the *psm-mec* gene (Kaito et al., 2011, 2013), in a way similar to the genetic layout of δ-toxin, whose gene is found within the regulatory RNA of the *agr* system, RNAIII (Novick, 2003). Interestingly, our group reported that the *psm-mec* srRNA transcript length was 143 bp, 14 nucleotides shorter than that described by Kaito et al. (2013) (Figure 1C). Notably, in any case, the *psm-mec* srRNA only barely exceeds the ORF coding for PSM-mec. This contrasts with RNAIII, which is considerably longer than the δ-toxin gene, *hld*. Additionally, the *psm-mec* srRNA is estimated to have a half-life of approximately 20 min (Kaito et al., 2013), which is significantly shorter than the ~45 min half-life described for RNAIII (Huntzinger et al., 2005). The only reported mechanism by which the *psm-mec* srRNA exerts it gene regulatory activity is through its interaction with the *agrA* transcript resulting in the overall decrease of AgrA activity (Kaito et al., 2013), which is entirely dependent on the first 60 nucleotides of the transcript (Kaito et al., 2013), where key anti-sense base-pair interactions occur (Cheung et al., 2014b). Because AgrA strictly regulates activity of the *psm*α and *psm*β promoters (Queck et al., 2008), many of the *psm-mec* srRNA-mediated phenotypes likely are mostly due to a repression of the production of the strongly cytolytic and pro-inflammatory PSMα peptides (Table 1). Kaito et al. initially observed up to eight-fold reduction in PSM expression when the *psm-mec* locus was over-expressed on a plasmid in methicillin-sensitive *S. aureus* strains or strains with SCC*mec* type IV (Kaito et al., 2011). However, the differences in PSM reduction were less dramatic, and often inconsistent, in isogenic *psm-mec* mutants naturally harboring SCC*mec* types II and III (Chatterjee et al., 2011), indicating a high degree of strain dependence regarding the role of the *psm-mec* srRNA in *S. aureus*.

**Table 1:** **Activities of the PSM-mec peptide and the p***sm-mec*** srRNA**.

**Virulence determinant within the *psm-mec* locus**	**Known *in vitro* activities in *S. aureus***
PSM-mec peptide	FPR2-mediated pro-inflammatory activity at nM concentrations: • Increased CD11b expression • Increased gp91phox expression • Increased IL-8 expression • Increased calcium flux • Increased chemotaxis
	Receptor-independent cytolytic activity at μM concentrations toward: • Erythrocytes • Neutrophils
	Peptide expression decreases oxacillin resistance^a^
*psm-mec* srRNA	Represses AgrA activity through direct interaction • Decreased PSM production^b^ • Enhanced biofilm formation and aggregation^b^ • Suppresses colony spreading • Up-regulates SpA expression^b^

a *Only tested in one background strain*.

b *Strain-specific phenotype*.

## The impact of the *psm-mec* srRNA on biofilm formation, colony spreading, and expression of *S. aureus* virulence factors

PSM peptides have a considerable impact on biofilm formation, which is based on their detergent-like properties (Vuong et al., 2000; Kong et al., 2006). PSMs cause the structuring and maturation of biofilms, which includes the formation of nutrient-transporting channels (Wang et al., 2011; Periasamy et al., 2012). PSMs also facilitate the detachment of biofilm clusters *in vitro* and *in vivo* (Wang et al., 2011; Periasamy et al., 2012; Otto, 2013b). It was shown that isogenic *agr* mutants (Vuong et al., 2000, 2004), total *psm* deletion mutants in all *psm* genes, and even single mutants in either the *psm*α, *psm*β, or *hld* loci (Wang et al., 2011; Periasamy et al., 2012) formed thicker biofilms compared to their wild type counterparts. Therefore, it was surprising to find that several isogenic *psm-mec S. aureus* mutants showed a reverse phenotype, albeit the change in biofilm formation was overall minor (Queck et al., 2009; Kaito et al., 2013). A phenotype of increased aggregation similar to that conferred by other PSMs (Dastgheyb et al., 2015) was only found in a strain in which the PSM-mec peptide was expressed in high relative amounts as compared to other PSMs (Queck et al., 2009). In most *psm-mec*-positive *S. aureus* strains, the regulatory effect of the *psm-mec* srRNA on the expression of other biofilm-dispersing PSMs (Periasamy et al., 2012) appears to over-shadow the lack of the direct dispersion effect of the PSM-mec peptide in *psm-mec* mutants (Kaito et al., 2013). Thus, in most strains, the impact of the *psm-mec* locus on biofilm formation is opposite to that of other *psm* loci, although that effect is generally not very pronounced.

For many pathogenic bacteria, motility plays an important role in colonization and virulence (Cossart, 2002; Josenhans and Suerbaum, 2002; Krukonis and DiRita, 2003). Motility is commonly dependent on the expression of flagella and type IV pili (Kearns, 2010). Even though *S. aureus* has always been historically regarded as non-motile, as it does not have the genes for flagella or pili, *S. aureus* has repeatedly been shown to spread efficiently across the surface of soft agar (Kaito et al., 2011; Tsompanidou et al., 2012) and more recently, on fresh pork meat (Tsompanidou et al., 2013), through a PSM-dependent mechanism (Lin et al., 2016), an effect now known as “colony-spreading” (Kaito and Sekimizu, 2007).

Originally, the observations of colony spreading and several other phenotypes were made using plasmid-based expression of the *psm-mec* locus in MRSA strains that did not carry the *psm-mec* locus, such as *S. aureus* isolates that were either methicillin-sensitive or MRSA strains that have recently emerged in the community (Community-Associated MRSA, CA-MRSA (Lowy, 1998; DeLeo et al., 2010). CA-MRSA strains are genetically distinct and notably more aggressive than HA-MRSA strains, show enhanced production of virulence factors, and are capable of causing disease in otherwise healthy individuals outside public healthcare settings (Tacconelli et al., 2004; Diep and Otto, 2008; Li et al., 2009; DeLeo et al., 2010). Furthermore, CA-MRSA strains characteristically possess SCC*mec* types IV or V (Diep and Otto, 2008; DeLeo et al., 2010) and therefore lack the *psm-mec* locus. Interestingly, CA-MRSA strains were reported to have a greater capacity to spread on soft agar compared to HA-MRSA strains (Kaito et al., 2008) and the absence of the *psm-mec* locus from CA-MRSA strains was speculated to cause that phenotype. Indeed, CA-MRSA strains transformed with plasmids containing the *psm-mec* locus were impaired in colony spreading. Conversely, isogenic HA-MRSA *psm-mec* mutants showed enhanced colony spreading (Kaito et al., 2013). Moreover, the inability of synthetic PSM-mec to rescue the colony spreading phenotype in an *agr* deletion strain of *S. aureus* (Tsompanidou et al., 2013) indicated that this regulation is predominantly mediated by the *psm-mec* srRNA. However, other studies showed that colony spreading is positively regulated by *agr* (Tsompanidou et al., 2011) and mainly controlled by mechanisms independent of the *psm-mec* srRNA, which include the production of several staphylococcal virulence factors, such as PSMs (Omae et al., 2012; Tsompanidou et al., 2013) and teichoic acids (Kaito and Sekimizu, 2007). In contrast, colony spreading is suppressed in the presence of multiple staphylococcal cell surface proteins (Tsompanidou et al., 2012). Taken together, the molecular mechanisms behind colony spreading in *S. aureus* reveal a somewhat complex network involving different, and often opposing, signals that affect the final phenotypic outcome. Lastly, it remains unknown whether colony spreading on agar surface is a mechanism universally adopted by all staphylococci and to what extent it matters for the colonization of epithelial surfaces *in vivo*.

In an effort to investigate *psm-mec*-dependent regulation in the natural strain background without artificial plasmid expression-induced effects, our group used microarray profiling to compare the transcriptomes of wild-type strains compared with those of isogenic *psm-mec* mutants (Cheung et al., 2014b). Most strikingly, the primary conserved target of *psm-mec*-dependent gene regulation was *spa*, the gene coding for the immune evasion protein, surface protein A (SpA), which was independently confirmed (Kaito et al., 2011). We also found that the strength of *psm-mec* srRNA transcription affected SpA expression (Cheung et al., 2014b). The up-regulation of SpA by the *psm-mec* srRNA is especially interesting because it adds to a number of existing transcriptional regulators known to control *spa* (Gao and Stewart, 2004). The up-regulated expression of SpA may play an important but yet unidentified role in the manifestation of staphylococcal disease (Gomez et al., 2004; Cheng et al., 2009; Merino et al., 2009; Kim et al., 2011) in some *psm-mec*-harboring *S. aureus* strains.

## The impact of the *psm-mec* locus on *S. aureus* virulence

Our group demonstrated that the ability of an isogenic *psm-mec S. aureus* mutant to form skin abscesses and cause sepsis in mouse models of infection was severely attenuated compared to the parent strain (Queck et al., 2009), indicating that the PSM-mec peptide contributed significantly to *S. aureus* pathogenesis. However, this phenotype could only be produced using an isogenic *psm-mec S. aureus* mutant whose parent strain produced more PSM-mec relative to other PSMs as compared to most *psm-mec*-harboring strains. Kaito et al. reported that the *psm-mec* srRNA contributes to virulence, but in a fashion opposite to that mediated by the PSM-mec peptide (Kaito et al., 2011, 2013). Furthermore, those authors proposed that the *psm-mec* srRNA was generally responsible for the differences in virulence between HA- and CA-MRSA strains (Kaito et al., 2013). They also speculated that point mutations in the *psm-mec* locus underlie increased virulence of a certain lineage, ST764, but this was not directly shown using isogenic mutants (Suzuki et al., 2016). However, a phenotype of increased virulence is not always observed in isogenic *psm-mec* HA-MRSA strains (Chatterjee et al., 2011), suggesting that the contribution of *psm-mec* srRNA toward *S. aureus* pathogenesis is strongly strain-dependent and cannot serve as a generally applicable explanation for differences in virulence between CA- and HA-MRSA strains.

## The roles of the PSM-mec peptide and the *psm-mec* regulatory RNA in other *Staphylococcus* species

In addition to *S. aureus*, the *psm-mec* locus is detected in many different methicillin-resistant *Staphylococcus* species (Monecke et al., 2012). However, PSM-mec expression levels have only been rigorously studied in *S. epidermidis* (Queck et al., 2009). Our group showed that PSM-mec expression was only detected in methicillin-resistant *S. epidermidis* infection isolates, but never in methicillin-sensitive *S. epidermidis* strains (Queck et al., 2009). There is no amino acid or nucleotide sequence difference (100% identity) between the *S. aureus* and *S. epidermidis* PSM-mec peptide or srRNA, respectively, based on a comparison of strains *S. aureus* MRSA252 and *S. epidermidis* RP62A, and differences are also absent or very minor in other strains. This is in accordance with the notion of a relatively recent move of SCC*mec* from coagulase-negative species to *S. aureus*.

To date, only one study has studied the impact of the *psm-mec* locus on the phenotypes in *S. epidermidis* and *S. haemolyticus* (Ikuo et al., 2014). The results, which were based on *S. epidermidis* and *S. haemolyticus* strains without SCC*mec* transformed with plasmids harboring the *psm-mec* locus, showed a decrease in PSM production, as demonstrated in similar experiments with *S. aureus* (Kaito et al., 2011). Intriguingly, an increase of biofilm formation was only observed in experiments with *S. epidermidis*, as reported previously (Kaito et al., 2011), but not *S. haemolyticus*, suggesting that the impact of the *psm-mec* locus on virulence phenotypes among staphylococci is also highly strain-dependent. The role of PSM-mec in coagulase-negative staphylococci has not yet been directly investigated using isogenic deletion mutants.

## Conclusion

It has become clear that the effects exerted by the *psm-mec* locus in *S. aureus* on virulence and virulence phenotypes such as biofilm formation are highly strain-dependent. They are also commonly rather moderate in extent compared to those conferred by other members of the PSM family. While great strides have been made to better understand the molecular underpinnings of gene regulatory *psm-mec*-dependent mechanisms in *S. aureus* pathogenesis, in the future the focus should be shifted toward the investigation of how the *psm-mec* locus affects the pathogenesis in other *Staphylococcus* species, especially *S. epidermidis* and *S. haemolyticus*, which, alongside *S. aureus*, are the most frequent and pathogenic agents of hospital-associated infections around the world. As it is a commonly accepted notion that SCC*mec* elements originate from coagulase-negative staphylococci (Otto, 2013c), such investigation is bound to shed light on the “original” role of the *psm-mec* locus in staphylococcal physiology, with the potential to also explain the contrasting roles of *psm-mec* in the *S. aureus* background.

## Author contributions

LQ, JM, GC, and MO contributed to the drafting of the manuscript and approved the final version.

## Funding

This work was supported by the Intramural Research Program of the National Institute of Allergy and Infectious Diseases, U.S. National Institutes of Health (grant ZIA AI000904-16).

### Conflict of interest statement

The authors declare that the research was conducted in the absence of any commercial or financial relationships that could be construed as a potential conflict of interest.
